# Giant Seebeck Effect in a PEDOT Material Coated on a Felt Fiber

**DOI:** 10.3390/ma18040838

**Published:** 2025-02-14

**Authors:** Hideki Arimatsu, Yuki Osada, Ryo Takagi, Takuya Fujima

**Affiliations:** Faculty of Science and Technology, Tokyo City University, Setagaya, Tokyo 158-8557, Japan; g1991001@tcu.ac.jp (H.A.);

**Keywords:** thermoelectric material, Seebeck coefficient, conductive polymer, PEDOT, molecular strain

## Abstract

Poly(3,4-ethylenedioxythiophene) (PEDOT) has been extensively investigated not only as a conductive polymer but also as a promising thermoelectric material. Numerous efforts have been undertaken to enhance the thermoelectric performance, particularly because improving the Seebeck coefficient is crucial for practical applications. In this study, we explored the thermoelectric property modification of PEDOT using a low-molecular carrier dopant and a fibrous substrate. PEDOT was coated on a felt texture with p-toluenesulfonic acid (PTSA) as the carrier dopant. The thermoelectric properties, including the Seebeck coefficient and electric conductivity, were measured. Raman spectroscopy was used to characterize the molecular strain of the PEDOT. The PEDOT sample coated on a felt texture with PTSA exhibited a wide range of Seebeck coefficients (−2100 to 3100 μV K^−1^). An estimation suggested the power factor reached 2400 µW m^−1^ K^−2^ for the p-type and 1100 µW m^−1^ K^−2^ for the n-type at the maxima. Raman spectroscopy showed a strong correlation between the strain in the C_β_-C_β_ bond of the PEDOT molecule and its Seebeck coefficient.

## 1. Introduction

Recently, the proliferation of IoT devices has been increasing the demand for autonomous power sources without depending on conventional centralized power sources from the standpoint of installation convenience and operational continuity, particularly during disasters [1,2,3]. Thermoelectric generation is expected to be an energy-harvesting technology that converts waste heat into electricity, and bismuth tellurium alloys have been put to practical use in the room-temperature range [4,5,6]. Organic thermoelectric materials are increasingly recognized as promising solutions for room-temperature thermoelectric applications owing to their flexibility, lightweight, and low toxicity. Various approaches are being explored for these materials’ practical application [7,8,9].

Thermoelectric materials are characterized by three properties: Seebeck coefficient (*S* [V K^−1^]), electric conductivity (*σ* [S m^−1^]), and thermal conductivity (*κ* [W m^−1^ K^−1^]). These are essential for evaluating the power factor (*S^2^σ*) and the figure of merit (*ZT* = *S^2^σTκ*^−1^), where *T* is the temperature. Typical thermoelectric modules are composed of p- and n-type thermoelectric conversion materials. The Seebeck coefficient is theoretically proportional to the differential value of the density of states (DOS) at the Fermi level (E_F_) with a negative constant.

Some organic compounds exhibit a significantly larger Seebeck effect than typical compounds with Seebeck coefficients on the order of 10^1^ µV K^−1^. In recent years, very large Seebeck coefficients, exceeding 0.1 V/K, have even been reported [10]. The mechanism for generating this large thermoelectric effect has been discussed from the perspective of phonon and carrier interaction [11,12,13,14,15].

A poly(3,4-ethylenedioxythiophene) (PEDOT)/polystyrene sulfonate (PSS) composite is a conductive polymer material that has been widely studied because of its high conductivity and chemical stability [16,17,18]. Herein, PSS provides a positive carrier for PEDOT and film stability, although it is intrinsically an insulator. We have also studied PEDOT films with high conductivity by using hierarchical nanoporous layer (HNL) glass to eliminate PSS [19] and by introducing a macro-separated structure of PEDOT and PSS [20]. 

PEDOT:PSS has emerged as a remarkable conductive polymer with exceptional versatility in flexible electronics and biomedical applications. This material exhibits outstanding properties, including tunable electrical conductivity up to more than 6300 S/cm, Ref. [21] excellent optical transparency in the visible spectrum, and high biocompatibility [22]. The stretchability of PEDOT:PSS can be significantly enhanced through various methods, such as blending with soft polymers or elastomers, achieving elongation rates exceeding 230% when combined with biocompatible polymers [23]. Recent advancements in PEDOT:PSS technology have expanded its applications in multiple fields, including flexible electronics, bioelectronics, energy storage systems, and tissue engineering. The development of novel micro/nanostructures and composite formations continues to push the boundaries of PEDOT:PSS capabilities [24,25].

PEDOT has also been studied as a thermoelectric material, but its typical Seebeck coefficient (a few tens of μV K^−1^) still requires further improvement for practical use [26]. This limitation is particularly crucial in the context of room-temperature applications, where PEDOT-based thermoelectric devices are most likely to be utilized. In these scenarios, the available temperature gradients are often small, making it challenging to generate sufficient voltage for powering even low-power electronic circuits. While voltage step-up technologies such as boost converters can be employed, they require a minimum input voltage to operate effectively. Consequently, substantially increasing the Seebeck coefficient of PEDOT materials is of paramount importance to achieve the necessary input voltage for these circuits.

In recent years, extensive research has been conducted to enhance the thermoelectric properties of PEDOT through various modification approaches. One common strategy involves creating composite materials by incorporating carbon-based materials such as carbon nanotubes, which has proven effective in improving electrical conductivity and thermoelectric performance [27]. Chemical post-treatment methods using ionic liquids have demonstrated significant enhancement of the power factor, with some treatments achieving values up to 239 μW m^−1^ K^−2^ [28]. Physical modifications have also been explored, with a femtosecond pulse laser to enhance its electrical conductivity [29].

PEDOT:PSS fibers have emerged as promising candidates for thermoelectric applications due to their unique properties such as mechanical flexibility and low thermal conductivity. These fibers are particularly advantageous for wearable energy harvesting applications. A continuous wet-spinning process followed by sulfuric acid treatment has been shown to significantly enhance the power factor of PEDOT:PSS fibers, achieving a power factor of 147.8 μW m^−1^ K^−2^, which is 15 times higher than that of two-dimensional films [30]. Similarly, the incorporation of tellurium nanowires (Te-NWs) into PEDOT:PSS fibers has resulted in a power factor of 385.4 μW m^−1^ K^−2^, attributed to the ordered alignment of nanowires and effective post-treatment processes [31]. The addition of polyvinyl alcohol and other polymers has been shown to enhance the mechanical properties without compromising electrical conductivity, further supporting their use in flexible applications [32].

In this study, we explored the use of a low-molecular carrier dopant, p-toluenesulfonic acid (PTSA), and a felt fiber as a fibrous substrate to modify the thermoelectric properties of PEDOT, especially the Seebeck coefficient. As a reference, commercial PEDOT:PSS was used because it has been used in many studies and is suitable as a benchmark [16,33]. The remainder of this paper is organized as follows. Section 2 introduces the preparation methods for PEDOT:PTSA coated on felt fabric and glass substrates, as well as reference samples using commercial PEDOT:PSS. Then, the characterization methods, including electrical resistivity and thermoelectromotive force measurements, are described. Section 3 shows the sample appearance and detail of the electrical resistivity, thermoelectromotive force, and Raman spectra of the samples. Section 4 provides an estimation of the volume conductivity of the PEDOT:PTSA/felt sample and its power factor and a discussion about the correlation between the Seebeck coefficient and molecular strain revealed by Raman spectroscopy. Section 5 gives the concluding remarks.

## 2. Materials and Methods

In this study, we used PEDOT supported on felt fibers without PSS but with p-toluenesulfonic acid (PTSA). Flat glass slides were used as substrates for comparison. PEDOT:PTSA was supported on the substrates (felt fabric [100% PET] and soda-lime glass) by immersing them in a PEDOT polymerization reaction solution. For the solution, 12 [μmol L^−1^] of 3,4-ethylenedioxytiophene (EDOT > 98.0%, Tokyo Chemical Industry Co., Ltd., Tokyo, Japan), 14 [mmol L^−1^] of sodium peroxyodisulfate (SPS > 97.0%, Fujifilm Wako Pure Chemicals Co., Ltd., Osaka, Japan), and 1.9 [mmol L^−1^] of p-Toluenesulfonic acid (PTSA > 99.0%, Fujifilm Wako Pure Chemicals Co., Ltd., Osaka, Japan) were mixed and dispersed in purified water. The substrates were kept in solution for 24 h at a controlled room temperature (23–25 °C) using a sealed pot to prevent water evaporation and then dried at 336 K for 12 h.

For PEDOT:PTSA samples supported on felt, also prepared variations by changing the PTSA concentration during polymerization. These samples were prepared under identical conditions to those described in the preceding paragraph, except for the PTSA concentration. Multiple samples were prepared with PTSA concentrations ranging from the previously mentioned 1.9 [mmol L^−1^] down to 0.19 [mmol L^−1^].

Samples with commercially available PEDOT:PSS (high-conductivity grade 0.5–1 wt%, Sigma Aldrich, St. Louis, MO, USA) were also prepared for comparison. PEDOT:PSS was attached onto the felt fabric through nine repetitions of immersion in the purchased PEDOT:PSS dispersion for 24 h and dried at 336 K for 12 h. The process was carried out at room temperature (23–25 °C) in a sealed pot. The dispersion was spin-coated onto a soda-lime glass.

Each sample was characterized using a scanning electron microscope (JCM−6000PLUS Neo Scope, JEOL Ltd., Tokyo, Japan), Raman scattering spectrometer (T64000, HORIBA Ltd., Kyoto, Japan) with an argon laser (514.5 nm), a wavenumber accuracy of 0.1 cm^−1^ and a wavenumber resolution of 0.15 cm^−1^. The Raman spectral data were analyzed using the Multipeak Fitting package integrated in Igor Pro 8 (8.04) (WaveMetrics Inc., Portland, OR, USA). Each spectrum was subjected to the fitting analysis using Voigt functions, where peak intensities, positions, and shape parameters were optimized without constraints.

Electrical measurements were performed using an LCR meter (ZM2376, NF Corp., Yokohama, Japan) and a digital multimeter (Model 2100 6½, Keithley Instruments, Solon, ME, USA). The LCR meter, featuring a basic accuracy of 0.08%, was employed for sheet resistivity evaluation via the four-terminal method. Thermoelectromotive force was measured using the Keithley digital multimeter, which offers a 6½-digit resolution with better than 0.01% basic DC voltage accuracy on the 100 mV range. The samples were sufficiently stable for several months to maintain their thermoelectric properties: Seebeck coefficient and sheet resistivity.

Peltier devices (VPE35–12–40S, VICS Corp., Tokyo, Japan) were used to control the temperature at both ends of the sample with 0.1 °C precision. Starting from an equilibrium state where both ends were at 303 K, the temperature difference was systematically varied by simultaneously increasing and decreasing the temperature of the hot and cold ends, respectively. The thermoelectromotive force was measured during both the increasing temperature difference process (0 to 10 K) and the subsequent decreasing process (10 to 0 K). At each measurement point, the temperatures at both ends were stabilized before recording the thermoelectromotive force. The temperature dependence of the thermoelectromotive force was measured four consecutive times, confirming the stability and consistency of the measurements through the reproducibility of the results.

## 3. Results

**Figure 1:**
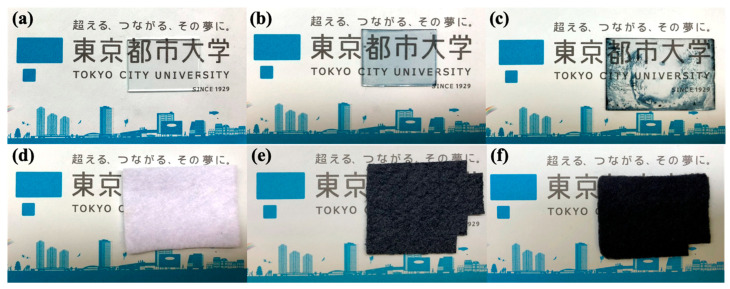
The appearance of samples using two substrates: (**a**–**c**) glass slide and (**d**–**f**) felt fabric. (**a**,**d**) Untreated substrates turned bluish both (**b**,**e**) with commercial PEDOT:PSS coated and (**c**,**f**) with PEDOT:PTSA coated.

The loading of the PEDOT turned the substrates dark blue after the preparation process, as shown in Figure 1. As PEDOT is a hydrophobic molecule, it adheres well to PET, which is also hydrophobic, but not to glass, which is hydrophilic. This results in the non-uniform adhesion of the PEDOT:PTSA/glass sample at the macroscopic level. Commercial PEDOT:PSS samples adhered well to glass because of the inclusion of the hydrophilic film-forming agent PSS. However, adhering to the felt fabric was difficult, requiring nine repetitions of the adhesion process described above.

**Figure 2:**
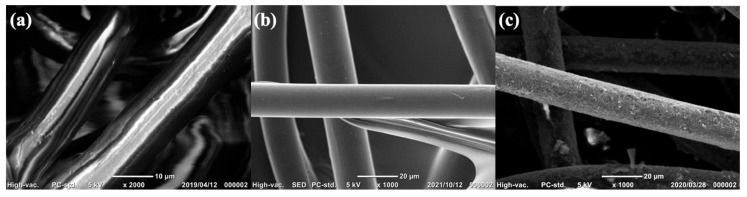
SEM micrographs of the felt fiber (**a**) without any treatment, (**b**) with PEDOT:PSS, and (**c**) with PEDOT:PTSA.

Figure 2 shows scanning electron micrographs of the samples featuring felt fabric substrates before and after the PEDOT loading treatment. Before the treatment, the image was prone to distortion owing to the absence of conductivity; however, both the PEDOT:PTSA and commercial PEDOT:PSS samples exhibited enhanced clarity, indicating their conductivity. The PEDOT materials adhered uniformly to the fibers.

**Figure 3:**
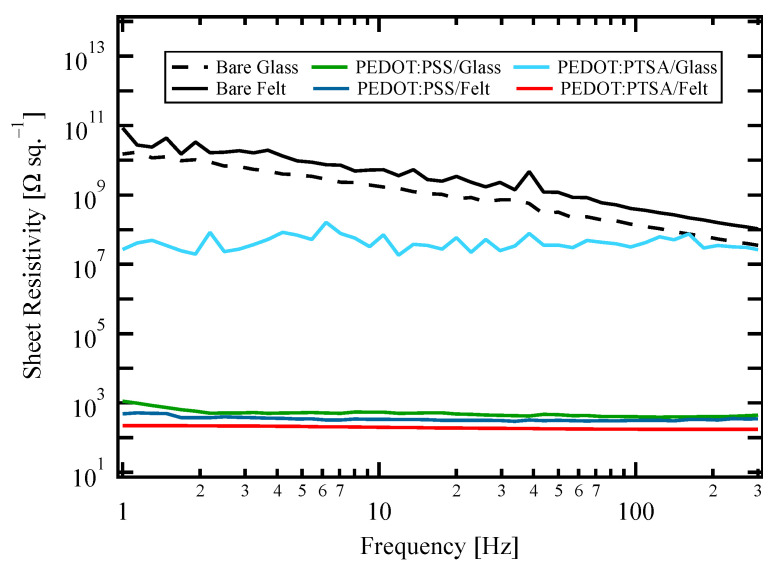
The sheet resistivity spectra of the samples.

The results presented in Figure 3 indicated the PEDOT provided conductivity for all samples. PEDOT:PTSA/glass had the highest resistivity, which can be due to the hydrophobic nature of PEDOT, making it challenging to adhere to the hydrophilic glass surface. In contrast, the other samples exhibited comparable low resistivities of approximately 200 Ω sq.^−1^ This suggests that the loading and doping of PEDOT on the felt fabric were comparable to those of commercial PEDOT:PSS of a high conductivity grade.

**Figure 4:**
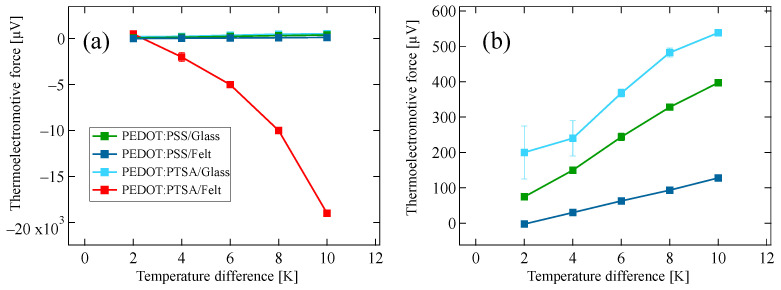
Thermoelectromotive force in PEDOT-coated samples as a function of temperature difference. (**a**) Comprehensive thermoelectric response for all samples. The PEDOT:PTSA/felt sample demonstrated a notably substantial voltage. (**b**) Enlarged view of the low-voltage region, elucidating details obscured in (**a**) due to scale.

Figure 4 shows the temperature gap dependence of the thermoelectromotive force. The PEDOT:PTSA/glass sample with a large resistivity and the commercial PEDOT:PSS samples exhibited values in the range of 10^1^ μV K^−1^, which are comparable to the values reported for general PEDOT materials. On the other hand, the PEDOT:PTSA/felt sample exhibited a larger Seebeck coefficient of −2100 μV K^−1^.

**Figure 5:**
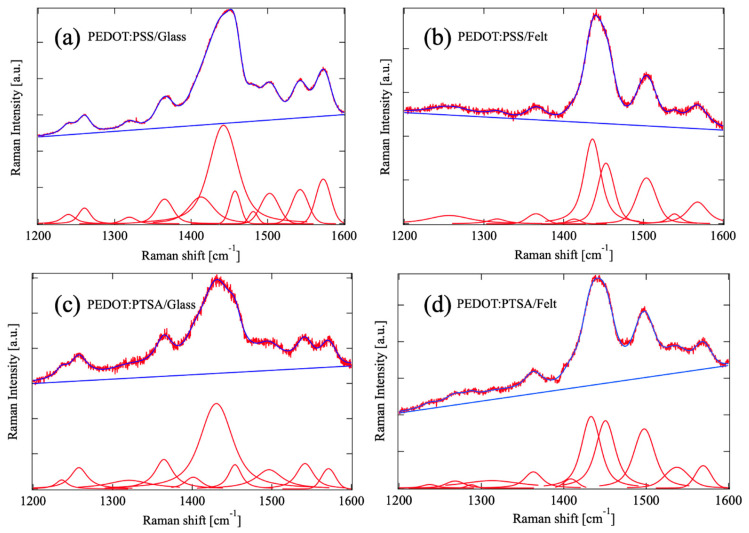
Raman spectra of (**a**) PEDOT:PSS on glass, (**b**) PEDOT:PSS on felt, (**c**) PEDOT:PTSA on glass, (**d**) PEDOT:PTSA on felt. Each spectrum (red line with noise) was reproduced (blue line along with the spectrum) with a straight baseline (blue) and Voigt peaks corresponding to chemical bonds in samples (red smooth lines).

The Raman spectra of the samples are shown in Figure 5. The obtained spectra were subjected to curve-fitting analysis using the peaks detected in PEDOT and the supporting materials (felt or glass) as Voigt functions. The baseline is assumed to be a straight line. As shown in the figure, the fitting analysis reproduced the experimental data well.

The peaks obtained through the analysis were assigned to specific chemical bonds within the PEDOT structure. The peaks at 1226 cm^−1^ and 1256–1270 cm^−1^ correspond to C_α_–C_α_ bonds, which serve as the connecting bonds between monomers, confirming successful polymerization. The peak around 1450 cm^−1^ is attributed to the symmetric vibration of C_α_=C_β_ bonds. This peak exhibits distinct positions for the undoped benzoidal structure and the doped quinoidal structure of the thiophene ring, providing insights into the doping state. The variations in the central wavenumbers of these assigned peaks yielded valuable information regarding the compression and elongation of their respective bonds.

**Figure 6:**
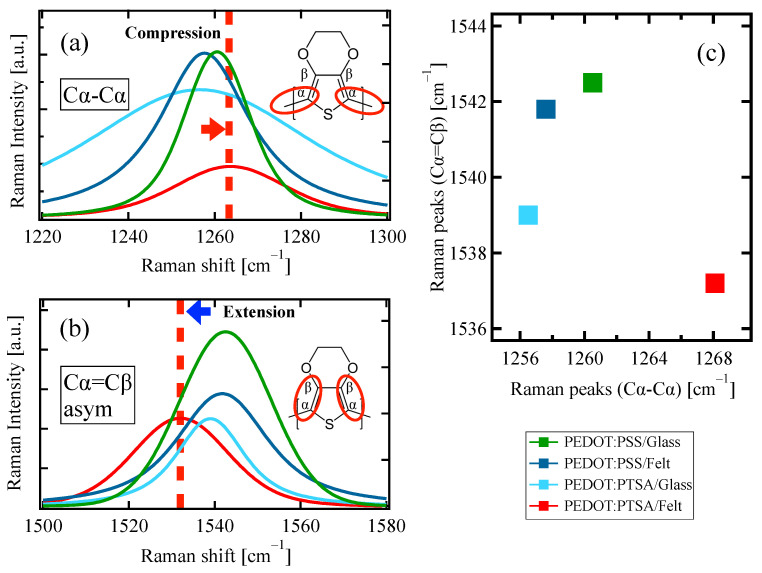
Raman scattering components for (**a**) C_α_-C_α_ bond, (**b**) C_α_=C_β_ asymmetric stretching, (**c**) correlation between the C_α_-C_α_ and the C_α_=C_β_ bonds.

Figure 6a,b show the specifics of the separated scattering peaks attributed to the interatomic bonds along the conduction path of the PEDOT chains. Figure 6c shows the correlation between the Raman shifts of the C_α_–C_α_ and C_α_=C_β_ bonds for each sample. This figure indicates that only the felt-supported PEDOT/PTSA had a significant difference in the molecular strain compared to the others.

**Figure 7:**
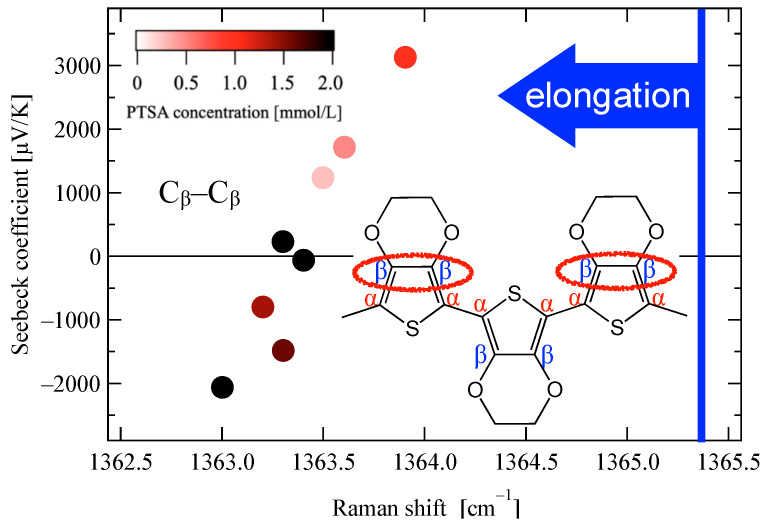
Seebeck coefficient of the PEDOT:PTSA sample on the felt fabric as a function of the Raman shift for the C_β_–C_β_ bond. The data points are color-coded according to the PTSA concentration in the PEDOT-polymerization solution, as indicated by the color scale in the graph. The solid blue line indicates the peak position of PEDOT:PSS/Glass.

The C_β_–C_β_ bond in the conduction path was strongly correlated with the Seebeck coefficient with a correlation coefficient of 0.91. As shown in Figure 7, varying the PTSA concentration in the polymerization solution resulted in a wide range of Seebeck coefficients for felt-supported PEDOT/PTSA. The values ranged up to significantly large values on the order of 10^3^ µV K^−1^ for both positive (3100 μV K^−1^) and negative (−2100 μV K^−1^) values. 

The lighter-colored plots representing lower PTSA concentrations exhibited smaller elongation of C_β_–C_β_ bonds, indicating structural characteristics closer to those of PEDOT:PSS. A general trend was observed where higher PTSA concentrations led to greater bond elongation across all conditions. This suggests that PTSA promotes the strain of C_β_–C_β_ bonds, indicating that the abundant presence of PTSA within the PEDOT material may act as a contributing factor in stretching these C_β_–C_β_ bonds.

## 4. Discussion

In the felt-supported samples, PEDOT was coated along the surface of each fine fiber, resulting in a three-dimensional intricate PEDOT film within the fabric. Consequently, assessing the intrinsic resistivity of PEDOT films is difficult due to their complex shapes and conduction paths. Here, we roughly estimated the volume conductivity of the PEDOT film by assuming that the PEDOT was homogeneously coated on the fibers, which were randomly and isotropically entangled to form an unwoven cloth. 

The diameter of the fibers was determined to be 17–20 µm through SEM observations, and the thickness of the PEDOT film on the fibers was 0.24–0.32 µm, as indicated by the mass difference before and after PEDOT loading. The volume-fill factor of the felt fabric was calculated to be 0.08 based on the difference between its apparent density and the commonly known density of PET. This resulted in an average of approximately 30 fibers stacked in the direction of fabric thickness. Consequently, the volume resistivity of PEDOT:PTSA on the felt was estimated to be approximately 4.0 mΩ m based on the sheet resistivity in Figure 3.

The obtained conductivity values are comparable to those of commercial PEDOT:PSS materials, suggesting potential for improvement through various post-treatments and hybridization methods that have been demonstrated in previous studies. The estimation of conductivity may be affected by several factors related to the sample structure. Non-uniform thickness of the PEDOT film within the sample could create bottlenecks in thinner regions or establish preferential conduction paths through thicker areas. While the microscopic observations assumed isotropic orientation and distribution of fibers within the felt, any directional bias could significantly alter the calculated values. It is important to note that these calculations represent a rough estimation based on macroscopically averaged assumptions of the sample structure.

The Raman scattering peak area ratios of the benzoidal and quinoidal C_α_=C_β_ symmetric vibrations were nearly identical in all the samples shown in Figure 7. This indicated that the doping levels were unaffected; thus, the change in the Seebeck coefficient in Figure 7 was not attributable to a difference in the carrier concentration.

The variation in the Seebeck coefficient between positive and negative values indicates that the slope of the density of states (DOS) at the Fermi level has reversed positive and negative; a peak or valley close to the Fermi level could traverse it within the range outlined in Figure 7. This modification could potentially be corroborated by first-principles calculations demonstrating a sharp impurity level in proximity to the Fermi level of PEDOT/PTSA [34]. 

The large Seebeck coefficients, both positive and negative, could be associated with a steep peak in the DOS. Within the range of Figure 7, as the C_β_–C_β_ bond is elongated, the peak should have moved from a position where the midpoint of the high-energy side overlapped the Fermi level to a position where the midpoint of the low-energy side overlapped. The molecular strain could potentially modify the boundary conditions for the wave function of the conductive carrier, leading to alterations in DOS, as reported for two-dimensional materials [35]. The strong correlation between the Seebeck coefficient and the elongation of the C_β_–C_β_ bond suggests a possible causality. However, at this stage, only the correlation has been definitively established, and further investigation would be necessary to elucidate the underlying mechanistic relationship between molecular distortion and thermoelectric properties.

This study realized both positive and negative thermoelectric properties within the same material system. Furthermore, the Seebeck coefficient values exceeded the previously reported maximum values by one order of magnitude for both positive and negative coefficients [36,37]. The estimated resistivity allows for approximate power factor determination. The maximum positive and negative Seebeck coefficients observed in this study yield 2400 µW m^−1^ K^−2^ and 1100 µW m^−1^ K^−2^, respectively, which are comparable to the highest power factor reported in the literature to date [38,39]. Determining the dimensionless figure of merit (ZT) poses a challenge because measuring the thermal conductivity of felt-supported samples is difficult due to their porosity.

## 5. Conclusions

The PEDOT/PTSA material coated on the PET fiber-made felt fabric demonstrated a considerable Seebeck effect. Specifically, the Seebeck coefficients for both the positive (3100 μV K^−1^) and negative (−2100 μV K^−1^) properties were significantly higher than the largest values reported to date. The power factor estimates were 2400 µW m^−1^ K^−2^ for the positive material and 1100 µW m^−1^ K^−2^ for the negative material. The strong correlation between the Seebeck coefficients and elongation of the C_β_–C_β_ bond within the PEDOT molecules suggests a possible relationship between the density of states (DOS) modification and molecular distortion.

The large Seebeck coefficient achieved in this study opens up possibilities for practical applications when combined with voltage step-up technologies, such as boost converters. The ability to fabricate both n- and p-type materials within the same material system represents a significant advantage, enabling the construction of practical thermoelectric units with improved electrical connectivity and substantial benefits in terms of manufacturing and maintenance. Looking ahead, we envision diverse applications of this technology. These include powering wearable devices and medical equipment using body heat as a thermal source, as well as developing ubiquitous power sources that harness temperature differentials between sunlit and shaded areas or between the ground and underground to achieve uninterrupted power supply for infrastructure. Although our findings regarding molecular strain correlation provide valuable insights, they remain phenomenological. Future research should focus on fully elucidating the mechanisms responsible for the enhanced power output and developing methods for precise control of these characteristics.

## 6. Patents

Based on the results of this research, a patent application has been filed in Japan. (Japanese Patent Application No. JP2021−172753)

## Data Availability

The original contributions presented in the study are included in the article; further inquiries can be directed to the corresponding author.

## Abbreviations

The following abbreviations are used in this manuscript:

PEDOT	poly(3,4-ethylenedioxythiophene)
PSS	polystyrene sulfonate
PTSA	p-toluenesulfonic acid
DOS	Density Of State

## References

[1] Espinoza O., Tiwary A. (2021). Assessment of Autonomous Renewable Energy System Operability under Extreme Events and Disasters. Sustain. Energy Technol. Assess..

[2] Masrur H., Gamil M.M., Islam M.R., Muttaqi K.M., Lipu M.S.H., Senjyu T. (2022). An Optimized and Outage-Resilient Energy Management Framework for Multicarrier Energy Microgrids Integrating Demand Response. IEEE Trans. Ind. Appl..

[3] Thirumalai M., Hariharan R., Yuvaraj T., Prabaharan N. (2024). Optimizing Distribution System Resilience in Extreme Weather Using Prosumer-Centric Microgrids with Integrated Distributed Energy Resources and Battery Electric Vehicles. Sustainability.

[4] Wei J., Yang L., Ma Z., Song P., Zhang M., Ma J., Yang F., Wang X. (2020). Review of Current High-ZT Thermoelectric Materials. J. Mater. Sci..

[5] Ang A.K.R., Yamazaki I., Hirata K., Singh S., Matsunami M., Takeuchi T. (2023). Development of Cu_2_ Se/Ag_2_ (S,Se)-Based Monolithic Thermoelectric Generators for Low-Grade Waste Heat Energy Harvesting. ACS Appl. Mater. Interfaces.

[6] Cui G.-P., Feng C.-P., Xu S.-C., Sun K.-Y., Ji J.-C., Hou L., Lan H.-B., Shang H.-J., Ding F.-Z. (2024). 3D-Printed Bi_2_ Te_3_-Based Thermoelectric Generators for Energy Harvesting and Temperature Response. ACS Appl. Mater. Interfaces.

[7] Bao Y., Sun Y., Jiao F., Hu W. (2023). Recent Advances in Multicomponent Organic Composite Thermoelectric Materials. Adv. Electron. Mater..

[8] Lee S., Kim S., Pathak A., Tripathi A., Qiao T., Lee Y., Lee H., Woo H.Y. (2020). Recent Progress in Organic Thermoelectric Materials and Devices. Macromol. Res..

[9] Zhang Y., Wang W., Zhang F., Dai K., Li C., Fan Y., Chen G., Zheng Q. (2022). Soft Organic Thermoelectric Materials: Principles, Current State of the Art and Applications. Small.

[10] Nakamura M., Kojima H., Abe R., Cho Y., Hayashi S., Hiramoto M. (2024). Giant Seebeck Effect over 0.1 V K^−1^—Is This an Intrinsic Phenomenon in Organic Semiconductors?. Faraday Discuss..

[11] Okada N., Sato K., Yokoo M., Kodama E., Kanehashi S., Shimomura T. (2021). Thermoelectric Properties of Poly(3-Hexylthiophene) Nanofiber Aerogels with a Giant Seebeck Coefficient. ACS Appl. Polym. Mater..

[12] Machida Y., Lin X., Kang W., Izawa K., Behnia K. (2016). Colossal Seebeck Coefficient of Hopping Electrons in (TMTSF) 2 PF 6. Phys. Rev. Lett..

[13] Takahashi H., Okazaki R., Ishiwata S., Taniguchi H., Okutani A., Hagiwara M., Terasaki I. (2016). Colossal Seebeck Effect Enhanced by Quasi-Ballistic Phonons Dragging Massive Electrons in FeSb_2_. Nat. Commun..

[14] Kojima H., Abe R., Fujiwara F., Nakagawa M., Takahashi K., Kuzuhara D., Yamada H., Yakiyama Y., Sakurai H., Yamamoto T. (2018). Universality of the Giant Seebeck Effect in Organic Small Molecules. Mater. Chem. Front..

[15] Chiang W.-H., Iihara Y., Li W.-T., Hsieh C.-Y., Lo S.-C., Goto C., Tani A., Kawai T., Nonoguchi Y. (2019). Enhanced Thermoelectric Properties of Boron-Substituted Single-Walled Carbon Nanotube Films. ACS Appl. Mater. Interfaces.

[16] Gueye M.N., Carella A., Faure-Vincent J., Demadrille R., Simonato J.-P. (2020). Progress in Understanding Structure and Transport Properties of PEDOT-Based Materials: A Critical Review. Prog. Mater. Sci..

[17] Liu L., Wu L., Yang H., Ge H., Xie J., Cao K., Cheng G., Chen S. (2022). Conductivity and Stability Enhancement of PEDOT:PSS Electrodes via Facile Doping of Sodium 3-Methylsalicylate for Highly Efficient Flexible Organic Light-Emitting Diodes. ACS Appl. Mater. Interfaces.

[18] Kim T., Park S., Seo J., Lee C.W., Kim J.-M. (2019). Highly Conductive PEDOT:PSS with Enhanced Chemical Stability. Org. Electron..

[19] Fujima T., Uchiyama K., Yasumoro K., Ito T., Tabata E. (2018). A PSS-Free PEDOT Conductive Film Supported by a Hierarchical Nanoporous Layer Glass. Macromol. Mater. Eng..

[20] Yasumoro K., Fujita Y., Arimatsu H., Fujima T. (2020). A New Composite Structure of PEDOT/PSS: Macro-Separated Layers by a Polyelectrolyte Brush. Polymers.

[21] Shi Y., Zhou Y., Shen R., Liu F., Zhou Y. (2021). Solution-Based Synthesis of PEDOT:PSS Films with Electrical Conductivity over 6300 S/Cm. J. Ind. Eng. Chem..

[22] Zhang X., Yang W., Zhang H., Xie M., Duan X. (2021). PEDOT:PSS: From Conductive Polymers to Sensors. Nanotechnol. Precis. Eng..

[23] Brendgen R., Grethe T., Schwarz-Pfeiffer A. (2024). Straightforward Production Methods for Diverse Porous PEDOT:PSS Structures and Their Characterization. Sensors.

[24] Zan G., Jiang W., Kim H., Zhao K., Li S., Lee K., Jang J., Kim G., Shin E., Kim W. (2024). A Core–Shell Fiber Moisture-Driven Electric Generator Enabled by Synergetic Complex Coacervation and Built-in Potential. Nat. Commun..

[25] Chen J., Ye C., Cang T., Gao R., Li X. (2023). Recent Advances in the Construction and Application of Stretchable PEDOT Smart Electronic Membranes. J. Mater. Chem. C.

[26] Xu Y., Jia Y., Liu P., Jiang Q., Hu D., Ma Y. (2021). Poly(3,4-Ethylenedioxythiophene) (PEDOT) as Promising Thermoelectric Materials and Devices. Chem. Eng. J..

[27] Stevens D.L., Gamage G.A., Ren Z., Grunlan J.C. (2020). Salt Doping to Improve Thermoelectric Power Factor of Organic Nanocomposite Thin Films. RSC Adv..

[28] Yemata T.A., Zheng Y., Kyaw A.K.K., Wang X., Song J., Chin W.S., Xu J. (2020). Improved Thermoelectric Properties and Environmental Stability of Conducting PEDOT:PSS Films Post-Treated With Imidazolium Ionic Liquids. Front. Chem..

[29] Wang X., Feng Y., Sun K., Chai N., Mai B., Li S., Chen X., Zhao W., Zhang Q. (2024). Ultrafast Laser-Induced Excellent Thermoelectric Performance of PEDOT:PSS Films. ENERGY Environ. Mater..

[30] Wen N., Fan Z., Yang S., Zhao Y., Cong T., Xu S., Zhang H., Wang J., Huang H., Li C. (2020). Highly Conductive, Ultra-Flexible and Continuously Processable PEDOT:PSS Fibers with High Thermoelectric Properties for Wearable Energy Harvesting. Nano Energy.

[31] Li J., Wang J., Yang X., Dong G., Liu Y., Wang Z., Zhang M., Zuo X., Han X., Wu C. (2024). Wet Spun Composite Fiber with an Ordered Arrangement of PEDOT:PSS-Coated Te Nanowires for High-Performance Wearable Thermoelectric Generator. Adv. Funct. Mater..

[32] Yang J., Jia Y., Liu Y., Liu P., Wang Y., Li M., Jiang F., Lan X., Xu J. (2021). PEDOT:PSS/PVA/Te Ternary Composite Fibers toward Flexible Thermoelectric Generator. Compos. Commun..

[33] Alhashmi Alamer F., Althagafy K., Alsalmi O., Aldeih A., Alotaiby H., Althebaiti M., Alghamdi H., Alotibi N., Saeedi A., Zabarmawi Y. (2022). Review on PEDOT:PSS-Based Conductive Fabric. ACS Omega.

[34] Arimatsu H., Osada Y., Takagi R., Fujima T. (2021). First-Principle Study on p-n Control of PEDOT-Based Thermoelectric Materials by PTSA Doping. Polymers.

[35] Zhang G., Zhang Y.-W. (2015). Strain Effects on Thermoelectric Properties of Two-Dimensional Materials. Mech. Mater..

[36] Li C., Shan C., Luo D., Gu X., Le Q., Kyaw A.K.K., Dong Z., Sun K., Ouyang J. (2024). Great Enhancement in the Seebeck Coefficient of PEDOT:PSS by Polaron Level Splitting via π–π Overlapping with Nonpolar Small Aromatic Molecules. Adv. Funct. Mater..

[37] Liu S., Li H., He C. (2019). Simultaneous Enhancement of Electrical Conductivity and Seebeck Coefficient in Organic Thermoelectric SWNT/PEDOT:PSS Nanocomposites. Carbon.

[38] Park T., Park C., Kim B., Shin H., Kim E. (2013). Flexible PEDOT Electrodes with Large Thermoelectric Power Factors to Generate Electricity by the Touch of Fingertips. Energy Environ. Sci..

[39] Choi K., Kim S.L., Yi S., Hsu J.-H., Yu C. (2018). Promoting Dual Electronic and Ionic Transport in PEDOT by Embedding Carbon Nanotubes for Large Thermoelectric Responses. ACS Appl. Mater. Interfaces.

